# How mobile are dye adsorbates and acetonitrile molecules on the surface of TiO_2_ nanoparticles? A quasi-elastic neutron scattering study

**DOI:** 10.1038/srep39253

**Published:** 2016-12-19

**Authors:** Valerie Vaissier, Victoria Garcia Sakai, Xiaoe Li, João T. Cabral, Jenny Nelson, Piers R. F. Barnes

**Affiliations:** 1 Department of Physcis, Imperial College London, London, SW72AZ, United Kingdom; 2 Centre for Plastics Electronics, Imperial College London, SW72AZ, United Kingdom; 3 ISIS Pulsed neutron and Muon Source, Rutherford Appleton Laboratory, Didcot, OX11 0QX, United Kingdom; 4 Department of Chemistry, Imperial College London, London, SW72AZ, United Kingdom; 5 Department of Chemical Engineering, Imperial College London, London, SW72AZ, United Kingdom

## Abstract

Motions of molecules adsorbed to surfaces may control the rate of charge transport within monolayers in systems such as dye sensitized solar cells. We used quasi-elastic neutron scattering (QENS) to evaluate the possible dynamics of two small dye moieties, isonicotinic acid (INA) and bis-isonicotinic acid (BINA), attached to TiO_2_ nanoparticles via carboxylate groups. The scattering data indicate that moieties are immobile and do not rotate around the anchoring groups on timescales between around 10 ps and a few ns (corresponding to the instrumental range). This gives an upper limit for the rate at which conformational fluctuations can assist charge transport between anchored molecules. Our observations suggest that if the conformation of larger dye molecules varies with time, it does so on longer timescales and/or in parts of the molecule which are not directly connected to the anchoring group. The QENS measurements also indicate that several layers of acetonitrile solvent molecules are immobilized at the interface with the TiO_2_ on the measurement time scale, in reasonable agreement with recent classical molecular dynamics results.

The properties of many materials can be tuned by exposing them to carefully chosen chemical functional groups. In particular, the functionalization of surfaces has extensive applications ranging from biology^1^,^2^,^3^,^4^ to molecular electronics^5^,^6^,^7^. For example, sensitizing semiconductor nanocrystals with dye molecules has led to the development of new types of solar fuel photoelectrodes^7^,^8^ and dye sensitized solar cells (DSSC)^9^,^10^. In these systems, the light absorbing properties of the dyes are combined with the electronic conductivity of the metal oxide substrate (often TiO_2_). However, the organic/inorganic interface created is often complex, making its properties difficult to predict. For example, the standard role for a dye molecule in a DSSC is to act as a centre for light absorption, then to inject the photoexcited electron into the TiO_2_ substrate, and to finally accept an electron from the surrounding electrolyte into the hole left on the dye following injection. However, in addition to these functions, lateral hole diffusion can occur in the dye monolayer by charge transfer between molecules anchored to adjacent sites on the TiO_2_ surface^11^,^12^,^13^,^14^,^15^,^16^,^17^,^18^,^19^,^20^. These interesting phenomena have been shown to improve the performance of solid state DSSCs^21^,^22^, and may play a role in recombination within liquid DSSCs and devices for water splitting^23^. Indeed, DSSCs can work without a separate hole transporting phase, with holes being collected solely via the dye monolayer^18^. Thus, identifying the factors controlling the kinetics of this process is of interest for the design of dye sensitized electronic devices.

We have recently shown that the kinetics of lateral hole diffusion appear to be influenced by the nature of the surrounding medium^17^,^19^,^24^, intermolecular interactions^20^, and – of particular relevance for this study – the structural rearrangement of anchored molecules at the surface^25^. Structural and vibrational dynamics of sensitized metal oxide surfaces have been investigated both theoretically with molecular dynamics^26^,^27^ and experimentally with X-ray, NMR, IR and photoelectron spectroscopy^28^,^29^,^30^,^31^,^32^. However, these techniques typically only probe the sub-ps timescale^33^,^34^. To be relevant to charge transport, we are interested in the timescale on which a charge transfer event occurs, typically 10^−8^ s or longer in these systems (dispersive transport is likely on a microscopic scale in these systems)^25^,^35^,^36^,^37^,^38^,^39^. This is much harder to characterize, due to both the static and dynamic disorder of the organic film on the surface^37^.

In this study we address the question: does the conformational rearrangement of dyes anchored to TiO_2_ occur on timescales comparable to hole hopping events? To investigate this problem, we used Quasi-Elastic Neutron Scattering (QENS) to probe sensitized TiO_2_ nanoparticles. The wavelength of the ‘thermal’ neutrons used and range of scattering angles allow us to investigate structures with length scales from 0.5 to 5.5 Å. We selected a back-scattering spectrometer that can detect changes in the scattered neutron energy corresponding to timescales between tens of ps and a few ns, spanning the relevant window.

As a model experimental system, we examined TiO_2_ nanoparticles (21 nm diameter) sensitized with a complete monolayer of one of two different dye moieties. These were isonicotinic acid (INA) which is a carboxylated pyridine, and the bipyridyl equivalent (BINA), which have one and two anchoring groups respectively (see Fig. 1)^28^. Since INA and BINA are not colored we will generally refer to them as adsorbates rather than dyes in this work. INA and BINA form the anchoring moieties in more complex monodentate dyes, such as indolene, and bidendate dyes, such as those based on ruthenium or cobalt. In selected experiments, the sensitized particles were immersed in acetonitrile since acetonitrile is commonly used as the solvent in DSSC electrolytes. The neutron scattering signals from the sensitized TiO_2_ samples, measured either as a dried powder, or combined with acetonitrile to make a paste, were compared to the scattering signal from unsensitized TiO_2_ nanoparticulate powder or paste. Since a significant proportion of the signal arises from the incoherent scattering contribution of hydrogen nuclei, analysis of the data should give information about the motion of individual molecules containing hydrogen such as the adsorbates.

Possible kinetics of the rotation of a dye’s body around the axis parallel to the TiO_2_ surface are illustrated in Fig. 1d. Due to the steric hindrance of the surrounding molecules, we expect that the adsorbates do not lie flat on the surface but are likely to adopt tilted configurations (blue lines in Fig. 1e) and may ‘flap’ between these tilted positions at some rate, *τ*^−1^. The dynamics of solvent itself could also influence the properties of the dye sensitized substrates^17^,^24^.

In the first section, we present experimental results that indicate that acetonitrile solvent molecules close to the TiO_2_ surface are likely to be immobilized within the measurement time window. These observations also serve as a partial control for the second section of the results where our measurements indicate that both INA and BINA anchored to the TiO_2_ nanoparticle surfaces are immobile within the time range of the measurement.

## Results

### Influence of the TiO_2_ on solvent (acetonitrile) diffusion

In this section we compare the scattering spectra obtained from pure hydrogenated acetonitrile (hACN) with that obtained from a paste of approximately 1:1 by weight of hACN and TiO_2_ nanoparticles. This allows us to test the possibility that the nanoparticles influence the diffusion of the ACN molecules.

**Figure 2:**
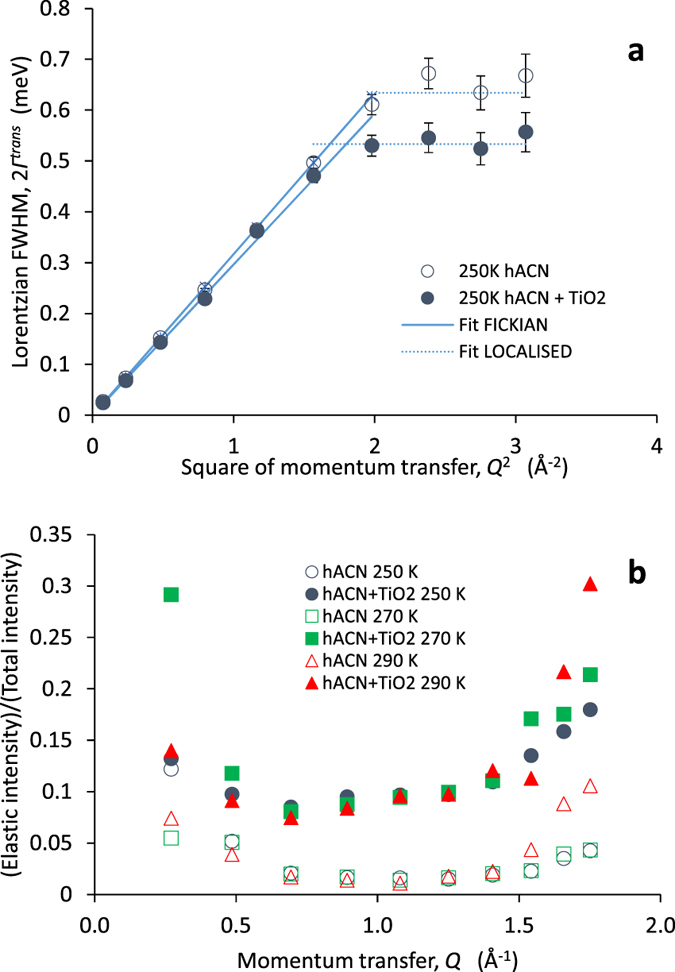
Analysis of QENS data from hydrogenated acetonitrile with and without TiO_2_ nanoparticles. (**a**) Full width half maximum (*Γ*^*trans*^) of the Lorentzian function used to fit the QENS data of pure hydrogenated acetonitrile (hACN) and the hACN + TiO_2_ nanoparticle paste plotted as a function of momentum transfer at 250 K. The *Γ*^*trans*^ values and uncertainties were determined from fits to the reduced data with the Dave package^48^. The linearly increasing section of the plot is fit by equation 1. The ‘plateau’ section at high *Q*^2^ is likely to correspond to the rotational motion of the CH_3_ groups of the acetonitrile^40^,^41^, whose timescale is 2ħ/*Γ*^*trans*^(plateau), approximately 2 ps (see Table S1). (**b**) Ratios of the area under the scattering intensity from the elastic peak to the total scattering intensity for hACN (open symbols) and hACN/TiO_2_ (closed symbols) plotted as a function of momentum transfer. The measurements were made at 250 (blue circles), 270 (red triangles) and 290 K.

The QENS spectra of pure acetonitrile could be well described by a self-diffusion model incorporating the translational motion of the ACN molecules^40^,^41^. Figure 2a shows an example of the full width half maximum (FWHM) of the Lorenzian function fitted to the broadening of the scattering peak attributed to translational motion of the hACN molecules (*Γ*^*trans*^) plotted against the square of momentum magnitude transferred, *Q*^2^, at 250 K (an example fit is shown in Figure S1).

For sufficiently low momentum transfer, the data are well described by a Fickian model of self-diffusion which predicts a linear dependence between the FWHM and *Q*^2^ in the limit of low momentum transfer:^40^,^42^


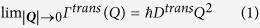


where *ħ* is Planck’s constant divided by 2π. The following self-diffusion coefficients are obtained at 250 K, 270 K and 290 K: *D*^*trans*^ = 2.41 ± 0.02 × 10^−5^ cm^2 ^s^−1^, 3.12 ± 0.05 × 10^−5^ cm^2 ^s^−1^ and 3.95 ± 0.07 × 10^−5^ cm^2 ^s^−1^ (see also Table S1 for the time constants corresponding to the plateaus at high *Q* values which can be interpreted in terms of expected methyl group rotation). The resulting Arrhenius activation energy of these values is 0.08 eV, enabling us to confirm by extrapolation that the diffusion coefficients we observe are consistent with those derived previously for acetonitrile at 298 K with QENS (*D*_298K_ = 4.2 ± 0.2 × 10^−5^ cm^2 ^s^−1^)^40^,^43^ and NMR (*D*_298K_ = 4.04 × 10^−5^ cm^2 ^s^−1^)^44^.

While the QENS spectra of the hACN + TiO_2_ nanoparticle paste indicated similar broadening to that of the pure hACN, the hACN diffusion coefficient in the paste was reduced by 5–10% by the presence of the TiO_2_ (Fig. 2a and Table S2). Comparison of the hACN + TiO_2_ with the pure hACN spectra also indicated a larger relative elastic contribution to the total scattering intensity in the presence of TiO_2_ (see Fig. 2b). Since only approximately 2% of the elastic scattering can be attributed to the presence of TiO_2_ (Table S2), this suggests that about 8% of the hACN maybe immobilized on the measurement timescale by the presence of the TiO_2_. We note that the minimum *Q* value detectable by the OSIRS instrument is 0.18 Å^−1^, and expect that if lower *Q* values were detectable then elastic scattering fraction would extrapolate to 1 at sufficiently low *Q*.

Acetonitrile has a surface density of approximately 24 Å^2^ per molecule^45^, so we infer the immobile fraction of the 1.6 g of solvent in the hACN + TiO_2_ sample corresponds to about 4.5 × 10^2 ^m^2^ assuming negligible evaporation during preparation. We estimate the exposed surface of the 1.5 g of 21 nm diameter TiO_2_ nanoparticles in the sample to be approximately 1.1 × 10^2 ^m^2^, a similar order of magnitude. This is consistent with the immobile fraction corresponding to around 3–4 immobile layers of acetonitrile molecules coating the TiO_2_ surface, with the remaining fraction experiencing close to bulk liquid diffusion. These observations are in reasonable agreement with first principles molecular dynamics simulations by Schiffman *et al*.^27^. who predicted the formation of several structured solvent layers at the TiO_2_ interface which suppress diffusion.

### Adsorbate dynamics

We now examine the possible dynamics of the dye moieties adsorbed on the TiO_2_ nanoparticles. Initially, we compare sensitized and unsensitized TiO_2_ without solvent. The uptake of INA and BINA was estimated from UV-vis measurements to be about 0.016 and 0.018 grams per gram of TiO_2_, respectively (1.6% and 1.8% by weight without solvent). This is consistent with complete coverage of the nanoparticles by a monolayer of the adsorbates.

**Figure 3:**
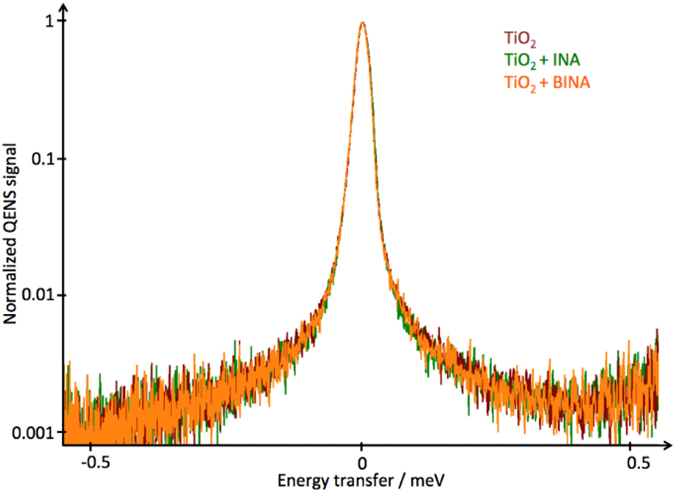
QENS spectra of sensitized TiO_2_ nanoparticles. QENS spectra of INA + TiO_2_ and BINA + TiO_2_ and TiO_2_ at 250 K summed over all detector angles, and normalized. No significant difference between the measurements can be observed. The measurements at 270 and 290 K also showed no variation between the spectra. After accounting for a slight peak broadening and background signal which we attribute to unavoidable contamination from adsorbed water species (observed in all samples containing TiO_2_) the spectra consist of only an elastic peak, indicating the scatterers are seen as immobile by OSIRIS.

A comparison of the QENS spectra for TiO_2_, TiO_2_ sensitized with INA, and TiO_2_ sensitized with BINA is shown in Fig. 3, indicating no significant difference in the spectra. This was the case for all *Q* groups at temperatures ranging from 250 to 290 K.

20% and 17% of the scattering signal in the sensitized samples should arise from incoherent contributions from INA and BINA respectively (see Table S1). Consequently, if the dye moieties were mobile within the spatio-temporal range probed by the measurement we would expect a measureable broadening of the elastic peak in the sensitized samples relative to the control sample of up to around 20% of the scattering intensity. Since no difference was observed, we conclude that INA and BINA do not move within the time window of the measurement, spanning approximately 10 ps and a few ns.

Next we examined whether the presence of acetonitrile surrounding the sensitized particles could allow motion of the moieties. To reduce the effect of incoherent scattering from hydrogen in the solvent which would mask any motion of the adsorbates, a 1:1 by weight ratio of deuterated acetonitrile (dACN) to BINA sensitized TiO_2_ particles was used. This was compared to a similar weight ratio of dACN and unsensitized TiO_2_. From Table S2 we expect incoherent scattering from BINA to contribute to only 5% of the total scattering signal in the sensitized sample with solvent. Thus even if BINA is mobile within the spatio-temporal window probed, we would expect its contribution to QENS measurement to be small and difficult to distinguish from the large contributions from dACN (77%) and TiO_2_ (18%). The magnitude of the QENS spectra from a pure dACN control sample was around 17% that of hACN, as expected due to the significantly lower incoherent scattering strength of deuterium relative to hydrogen. A comparison of QENS spectra of the mixture of dACN + BINA + TiO_2_, dACN + TiO_2_ and dACN is shown Figure S2.

**Figure 4:**
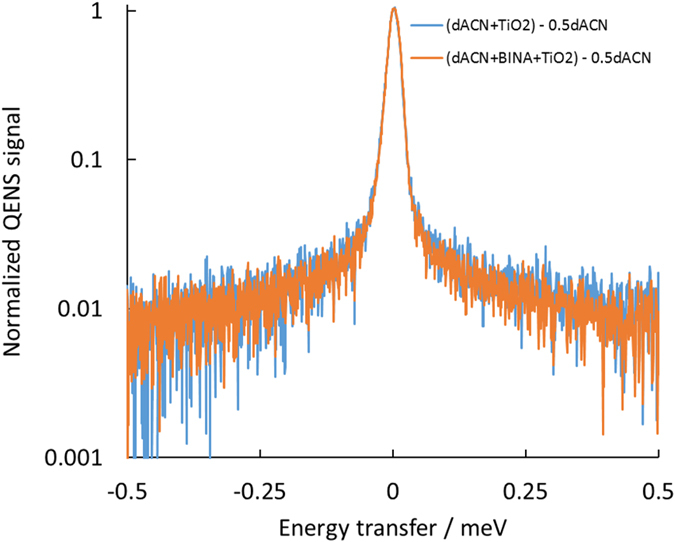
Scattering spectra from the dACN + BINA + TiO_2_ and dACN + TiO_2_ corrected for the expected contribution from dACN. QENS signal summed for all detector angles are plotted. The spectra correspond to the scattering from each sample after subtracting the spectrum from pure dACN weighted to correspond to 50% by mass of the sample. No significant difference can be discerned between the sample with and without BINA.

In an effort to remove the contribution from dACN to these data, the scattering spectrum of pure dACN multiplied by its expected contribution (inferred from its weight) in the sample was subtracted from the spectrum of the sample mixtures. The resulting spectra were then normalized. As above, no appreciable difference can be seen between the sensitized and unsensitized mixtures (see Fig. 4). Thus, given the experimental uncertainty, we see no evidence for the motion of BINA within the spatio-temporal window probed between 270 and 290 K, which is consistent with the observations from the INA and BINA sensitized samples in the absence of solvent.

## Discussion

We observed that TiO_2_ nanoparticles immersed in acetonitrile immobilise a fraction of acetonitrile molecules corresponding to several monolayer coverage of the particles. The remaining mobile fraction experiences close to bulk diffusion, consistent with previous computational work^27^. We believe that polar solvents (such as acetonitrile) will result in a strong interaction with the TiO_2_ surface leading most molecules in contact with the surface being immobilised. Apolar/aprotic solvent are less likely to form completly immobilized interfacial layers. It is possible that the adsorption of dye molecules to the TiO_2_ surface might reduce the immobilisation acetonitrile however we did not have a straightforward approach to distinguish between these two immobilized components with our experiments.

The monolayers of the dye moieties, INA and BINA, anchored to the TiO_2_ were immobile on the timescale resolvable with OSIRIS (tens of picoseconds to few nanoseconds). If structural rearrangements of these molecules occur, they do so on longer timescales. Extrapolation of our observations to larger dyes suggests that molecules containing anchoring moieties similar to INA and BINA, packed on the TiO_2_ surface with a similar coverage, are unlikely to undergo significant motions articulated from the anchoring point within this window. Clearly, more complex dye molecules could allow additional degrees of freedom around which motion of other moieties are be possible. This would be necessary (on timescales exceeding nanoseconds, possibly up to microseconds) if changes in molecular conformation explain the observed charge diffusion rates^25^. A wider temporal resolution and smaller *Q***-**range would be necessary to resolve slower and rotational relaxations of larger molecules. Other techniques such as nuclear magnetic resonance relaxation might also yield information on the possible molecular dynamics on longer timescales^46^.

## Methods

Sample preparation: 1.2 grams of 2,2′-Bipyridyl-4,4′-dicarboxylic acid (BINA) was added to 400 mL of N,N-Dimethyl formamide (DMF) in a 500 mL bottle. Alternatively, 1.2 grams of Pyridine-4-carboxylic acid (INA) was added to 400 mL ethanol. The solution was then heated to 70 °C and stirred at 700–800 rpm until complete dissolution of the dye. 3 grams of Titanium (IV) oxide (TiO_2_) anatase nanopowder (∼21 nm particle size, Sigma Aldrich) was added to the solution. The suspension was centrifuged at 4000 rpm for 5 minutes, followed by three ethanol washes, each followed by further centrifugation at 4000 rpm for 5 minutes to ensure that no residue of the high boiling point DMF (*T*_*boil*_(MeOH) = 153 °C) remained in the sample. The mass of dye adsorbed to the TiO_2_ was determined by UV-vis spectrophotometry of the dye solution before and after the addition of the nanoparticles. The samples were stored in ethanol overnight. Just before the loading of the sample cans, the samples were dried in two steps: hotplate at 80 °C and vacuum oven at 80 °C (*T*_*boil*_(MeOH) = 78 °C) for approximately half an hour each. Finally, about 1.5 grams of dried powder was either transferred into the same mass of solvent (hydrogenated or deuterated acetonitrile) or directly into the sample sachet made of aluminium foil. For the samples with solvent, the resulting paste was spread onto the sachet. The sachets were rolled into the OSIRIS cylindrical sample cans made of aluminium where the sample was contained within an annulus 0.5–1 mm thick with a circumference of around 70 mm and height 50 mm.

Quasi-elastic neutron scattering (QENS) measurements were performed at OSIRIS, a high resolution (25 μ eV) spectrometer within the ISIS pulsed neutron source facilities in the Rutherford Appleton Laboratory (Oxfordshire). The dynamic energy range and momentum transfer range (*Q*-range) are −0.4–0.4 meV and 0.18–1.8 Å^−1^ respectively. The analysing energy is 1.84 meV. Calibration measurements were made using a purely incoherent scattering vanadium sample. Including controls, measurements were performed on samples of pure hydrogenated acetonitrile, pure deuterated acetonitrile, TiO_2_ nanoparticle powder, TiO_2_ nanoparticle paste with hydrogenated acetonitrile, TiO_2_ nanoparticle paste with deuterated acetonitrile, INA or BINA sensitized TiO_2_ nanoparticles, and BINA sensitized TiO_2_ nanoparticle paste with deuterated acetonitrile. All the aforementioned measurements were made at three temperatures 250, 270 and 290 K which are above the melting point of acetonitrile (228 K). The beam intensity at the sample was 2.7 × 10^7^ neutrons cm^−2^ s^−1^ at 150 μA ISIS beam current, and samples were measured for 30 μAh.

The QENS spectra *S*_*inc*,*H*_(*Q, E′*), for each sample were extracted from the raw QENS data (*I*_*sc*_) with the software package Mantid (data reduction)^47^. The data from different ranges of detector angles were combined into ten groups to improve the measurement statistics.

The possible dynamics of molecules can be resolved by analysing the intensity of the spectrum of scattered neutrons from each nuclei, *S*_*i,j*_, which parametrically depends on the scattering regime *i* (i.e. coherent or incoherent) and the type of the nuclei in the sample *j. S*_*i,j*_ is related to the intensity of the scattered neutron beam, *I*_*sc*_, through:^41^,^42^


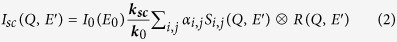


where *I*_*0*_ is the incident neutron flux, (*E*_*0*_, ***k***_***0***_) and (*E′, **k***_***sc***_) are the incident and scattered neutron beam energy and wavevector respectively, *Q* is the magnitude of the momentum transfer (*Q* =| ***k***_***sc***_ **−** ***k***_***0***_|) and *α*_*i,j*_ a factor accounting for the relative scattering magnitude of each nucleus *j* in regime *i*. The scattered signal is convolved with the instrument response function, *R*(*Q, E′*), which results in a spread of energies for all scattering events. For coherent scattering, 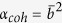 while for incoherent scattering, 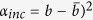 where *b* and 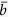 are the scattering length and spatially averaged scattering length of each nuclei^41^,^42^. These scattering lengths were used to calculate the expected contributions to the total scattering signal from the different samples examined in this study (Table S2). For hydrogenated samples, such as acetonitrile (hydrogenated ACN was used for measurements of solvent diffusion), INA and BINA, a major contribution to the total intensity is the incoherent scattering signal of hydrogen^40^,^41^,^42^. Consequently, the scattering intensity can often be approximated by:


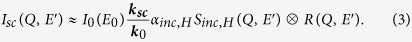


However, due to the low concentration of the dye moieties in this study the contribution to the scattering spectrum from the other nuclei was often significant. The relative contribution to the scattering signal from each component in the different samples is shown in Table S2, this was calculated from the scattering cross sections and molar fraction of the different nuclei in the samples.

## Additional Information

**How to cite this article**: Vaissier, V. *et al*. How mobile are dye adsorbates and acetonitrile molecules on the surface of TiO_2_ nanoparticles? A quasi-elastic neutron scattering study. *Sci. Rep.* **6**, 39253; doi: 10.1038/srep39253 (2016).

**Publisher's note:** Springer Nature remains neutral with regard to jurisdictional claims in published maps and institutional affiliations.

## Supplementary Material

Supplementary Information

## Figures and Tables

**Figure 1:**
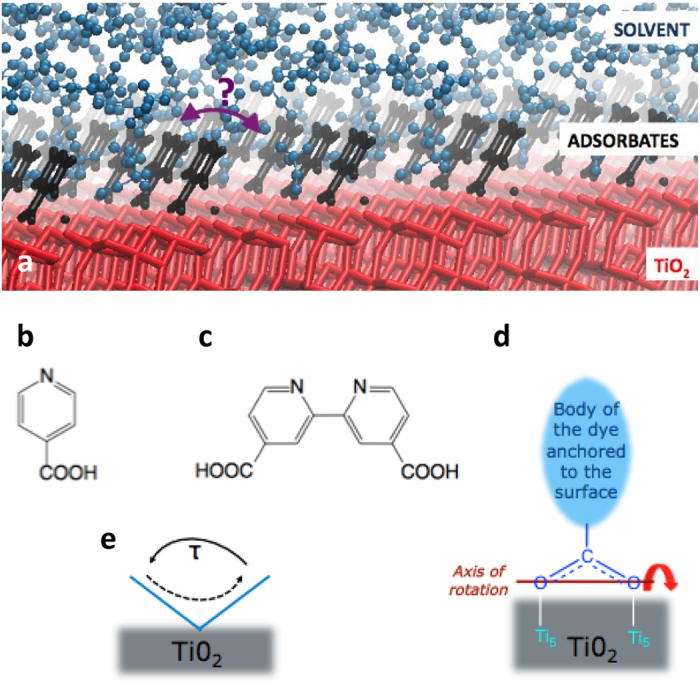
Dye moieties anchored to TiO_2_ surface and possible movements. (**a**) Schematic of the acetonitrile/INA/TiO_2_ interface. Chemical structure of (**b**) INA and (**c**) BINA, model for dye molecules with one and two anchoring groups respectively. (**d**) Possible rotational relaxation probed in this work: dye molecule rotation anchored to the metal oxide surface and (**e**) side view. The dye may explore conformations bounded by tilted positions (blue line) with a characteristic time constant *τ*.

## References

[b1] Alekhin A. P. . Synthesis of biocompatible surfaces by nanotechnology methods. Nanotechnologies in Russia 5, 696–708, doi: 10.1134/s1995078010090144 (2010).

[b2] Bruce I. J. & Sen T. Surface Modification of Magnetic Nanoparticles with Alkoxysilanes and Their Application in Magnetic Bioseparations. Langmuir 21, 7029–7035, doi: 10.1021/la050553t (2005). 16008419

[b3] Chu P. K., Chen J. Y., Wang L. P. & Huang N. Plasma-surface modification of biomaterials. Materials Science and Engineering: R: Reports 36, 143–206, doi: 10.1016/S0927-796X(02)00004-9 (2002).

[b4] Qin M. . Two methods for glass surface modification and their application in protein immobilization. Colloids and Surfaces B: Biointerfaces 60, 243–249, doi: 10.1016/j.colsurfb.2007.06.018 (2007). 17681764

[b5] Roelofs K. E., Brennan T. P. & Bent S. F. Interface Engineering in Inorganic-Absorber Nanostructured Solar Cells. Journal of Physical Chemistry Letters 5, 348–360, doi: 10.1021/jz4023656 (2014). 26270710

[b6] Li Y., Lu Q., Qian X., Zhu Z. & Yin J. Preparation of surface bound silver nanoparticles on polyimide by surface modification method and its application on electroless metal deposition. Applied Surface Science 233, 299–306, doi: 10.1016/j.apsusc.2004.03.235 (2004).

[b7] Sun B. & Skyllas-Kazacos M. Chemical modification of graphite electrode materials for vanadium redox flow battery application—part II. Acid treatments. Electrochimica Acta 37, 2459–2465, doi: 10.1016/0013-4686(92)87084-D (1992).

[b8] Zhao Y. . Improving the efficiency of water splitting in dye-sensitized solar cells by using a biomimetic electron transfer mediator. Proceedings of the National Academy of Sciences 109, 15612–15616, doi: 10.1073/pnas.1118339109 (2012). PMC3465399 22547794

[b9] O’Regan B. & Gratzel M. A low-cost, high efficiency solar-cell based on dye-sensitized colloidal TiO2 films. Nature 353, 737–740 (1991).

[b10] Bach U. . Solid-state dye-sensitized mesoporous TiO2 solar cells with high photon-to-electron conversion efficiencies. Nature 395, 583–585 (1998).

[b11] Bonhôte P. . Efficient Lateral Electron Transport inside a Monolayer of Aromatic Amines Anchored on Nanocrystalline Metal Oxide Films. The Journal of Physical Chemistry B 102, 1498–1507, doi: 10.1021/jp972890j (1998). 27577008

[b12] Trammell S. A. & Meyer T. J. Diffusional Mediation of Surface Electron Transfer on TiO2. Journal of Physical Chemistry B 103, 104–107, doi: 10.1021/jp9825258 (1999).

[b13] Wang Q. . Cross Surface Ambipolar Charge Percolation in Molecular Triads on Mesoscopic Oxide Films. Journal of the American Chemical Society 127, 5706–5713, doi: 10.1021/ja0426701 (2005). 15826212

[b14] Yang L. . Comparing spiro-OMeTAD and P3HT hole conductors in efficient solid state dye-sensitized solar cells. Phys. Chem. Chem. Phys. 14, 779–789, doi: 10.1039/c1cp23031j (2012). 22116450

[b15] Fillinger A. & Parkinson B. A. The Adsorption Behavior of a Ruthenium‐Based Sensitizing Dye to Nanocrystalline TiO2 Coverage Effects on the External and Internal Sensitization Quantum Yields. Journal of the Electrochemical Society 146, 4559–4564, doi: 10.1149/1.1392674 (1999).

[b16] Ardo S. & Meyer G. J. Direct Observation of Photodriven Intermolecular Hole Transfer across TiO2 Nanocrystallites: Lateral Self-Exchange Reactions and Catalyst Oxidation. Journal of the American Chemical Society 132, 9283–9285, doi: 10.1021/ja1035946 (2010). 20565127

[b17] Moia D. . The reorganization energy of intermolecular hole hopping between dyes anchored to surfaces. Chemical Science 5, 281–290 (2014).

[b18] Moia D. . Dye Monolayers Used as the Hole Transporting Medium in Dye-Sensitized Solar Cells. Advanced Materials 27, 5889–5894, doi: 10.1002/adma.201501919 (2015). 26308374

[b19] Manke F., Frost J. M., Vaissier V., Nelson J. & Barnes P. R. F. Influence of a nearby substrate on the reorganization energy of hole exchange between dye molecules. Phys. Chem. Chem. Phys. 17, 7345–7354, doi: 10.1039/C4CP06078D (2015). 25697305

[b20] Vaissier V., Frost J. M., Barnes P. R. F. & Nelson J. Influence of Intermolecular Interactions on the Reorganization Energy of Charge Transfer between Surface-Attached Dye Molecules. Journal of Physical Chemistry C 119, 24337–24341, doi: 10.1021/acs.jpcc.5b09739 (2015).

[b21] Moia D. . The Role of Hole Transport between Dyes in Solid-State Dye-Sensitized Solar Cells. The Journal of Physical Chemistry C 119, 18975–18985, doi: 10.1021/acs.jpcc.5b05222 (2015).

[b22] Weisspfennig C. T. . Dependence of Dye Regeneration and Charge Collection on the Pore-Filling Fraction in Solid-State Dye-Sensitized Solar Cells. Adv. Funct. Mater. 24, 668–677, doi: 10.1002/adfm.201301328 (2014).

[b23] Nattestad A. . Highly efficient photocathodes for dye-sensitized tandem solar cells. Nat Mater 9, 31–35, doi: 10.1038/nmat2588 (2010). 19946281

[b24] Vaissier V., Barnes P., Kirkpatrick J. & Nelson J. Influence of polar medium on the reorganization energy of charge transfer between dyes in a dye sensitized film. Phys. Chem. Chem. Phys. 15, 4804–4814, doi: 10.1039/c3cp44562c (2013). 23439984

[b25] Vaissier V. . Effect of Molecular Fluctuations on Hole Diffusion within Dye Monolayers. Chemistry of Materials 26, 4731–4740, doi: 10.1021/cm502629c (2014).

[b26] Pastore M. & De Angelis F. Aggregation of Organic Dyes on TiO2 in Dye-Sensitized Solar Cells Models: An ab Initio Investigation. Acs Nano 4, 556–562, doi: 10.1021/nn901518s (2010). 20020758

[b27] Schiffmann F., Hutter J. & VandeVondele J. Atomistic simulations of a solid/liquid interface: a combined force field and first principles approach to the structure and dynamics of acetonitrile near an anatase surface. Journal of Physics: Condensed Matter 20, 064206 (2008). 21693868 10.1088/0953-8984/20/6/064206

[b28] Thomas A. G. . Adsorption of bi-isonicotinic acid on anatase TiO2(1 0 1) and (0 0 1) studied by photoemission and NEXAFS spectroscopy. Surface Science 592, 159–168, doi: 10.1016/j.susc.2005.07.013 (2005).

[b29] Yella A. . Porphyrin-Sensitized Solar Cells with Cobalt (II/III)–Based Redox Electrolyte Exceed 12 Percent Efficiency. Science 334, 629–634, doi: 10.1126/science.1209688 (2011). 22053043

[b30] Manias E. & Kuppa V. The origins of fast segmental dynamics in 2 nm thin confined polymer films. Eur. Phys. J. E 8, 193–199, doi: 10.1140/epje/i2001-10074-x (2002). 15010968

[b31] Blanchet V., Zgierski M. Z., Seideman T. & Stolow A. Discerning vibronic molecular dynamics using time-resolved photoelectron spectroscopy. Nature 401, 52–54 (1999).

[b32] Zax D. B. . Dynamical heterogeneity in nanoconfined poly(styrene) chains. Journal of Chemical Physics 112, 2945–2951, doi: 10.1063/1.480867 (2000).

[b33] Laenen R., Simeonidis K. & Laubereau A. Subpicosecond Spectroscopy of Liquid Water in the Infrared: Effect of Deuteration on the Structural and Vibrational Dynamics. The Journal of Physical Chemistry B 106, 408–417, doi: 10.1021/jp011047p (2002).

[b34] Parikh A. N. & Allara D. L. Quantitative determination of molecular structure in multilayered thin films of biaxial and lower symmetry from photon spectroscopies. I. Reflection infrared vibrational spectroscopy. Journal of Chemical Physics 96, 927–945, doi: 10.1063/1.462847 (1992).

[b35] Druger S. D., Nitzan A. & Ratner M. A. Dynamic bond percolation theory: A microscopic model for diffusion in dynamically disordered systems. I. Definition and one‐dimensional case. Journal of Chemical Physics 79, 3133–3142, doi: 10.1063/1.446144 (1983).

[b36] Troisi A. & Orlandi G. Dynamics of the Intermolecular Transfer Integral in Crystalline Organic Semiconductors. Journal of Physical Chemistry A 110, 4065–4070, doi: 10.1021/jp055432g (2006). 16539430

[b37] Vehoff T. . Charge Transport in Self-Assembled Semiconducting Organic Layers: Role of Dynamic and Static Disorder. Journal of Physical Chemistry C 114, 10592–10597, doi: 10.1021/jp101738g (2010).

[b38] Troisi A., Ratner M. A. & Zimmt M. B. Dynamic Nature of the Intramolecular Electronic Coupling Mediated by a Solvent Molecule: A Computational Study. Journal of the American Chemical Society 126, 2215–2224, doi: 10.1021/ja038905a (2004). 14971957

[b39] Ciuchi S., Fratini S. & Mayou D. Transient localization in crystalline organic semiconductors. Physical Review B 83, 081202 (2011). 10.1103/PhysRevLett.106.166403 21599392

[b40] Kunz W., Turq P., Bellissent‐Funel M. C. & Calmettes P. Dynamics and spatial correlations of tetrapentylammonium ions in acetonitrile. Journal of Chemical Physics 95, 6902–6910, doi: 10.1063/1.461502 (1991).

[b41] Kunz W., Calmettes P. & Bellissent‐Funel M. C. Dynamics of liquid acetonitrile at high frequencies. Journal of Chemical Physics 99, 2079–2082, doi: 10.1063/1.465273 (1993).

[b42] Bee M. J. Quasielastic neutron scattering. Principles and Applications in Solid State Chemistry. (Adam Hilger, 1987).

[b43] Samigullin F. M. Study of translational self-diffusion of molecules in liquids. Journal of Structural Chemistry 14, 569–574, doi: 10.1007/bf00743282.

[b44] Kovacs H., Kowalewski J., Maliniak A. & Stilbs P. Multinuclear relaxation and NMR self-diffusion study of the molecular dynamics in acetonitrile-chloroform liquid mixtures. Journal of Physical Chemistry 93, 962–969, doi: 10.1021/j100339a080 (1989).

[b45] Wang H. . Molecules at Liquid and Solid Surfaces. Langmuir 14, 1472–1477, doi: 10.1021/la9707179 (1998).

[b46] Sapienza P. J. & Lee A. L. Using NMR to study fast dynamics in proteins: methods and applications. Current opinion in pharmacology 10, 723–730, doi: 10.1016/j.coph.2010.09.006 (2010). 20933469 PMC3001252

[b47] Arnold O. . Mantid—Data analysis and visualization package for neutron scattering and SR experiments. Nuclear Instruments and Methods in Physics Research Section A: Accelerators, Spectrometers, Detectors and Associated Equipment 764, 156–166, doi: 10.1016/j.nima.2014.07.029 (2014).

[b48] Azuah R. T. . DAVE: A comprehensive software suite for the reduction, visualization, and analysis of low energy neutron spectroscopic data. J. Res. Natl. Inst. Stan. Technol. 114, 341 (2009). 10.6028/jres.114.025 PMC4646530 27504233

