# Supplementary material for: How mobile are dye adsorbates and acetonitrile molecules on the surface of TiO2 nanoparticles? A quasi-elastic neutron scattering study
Source: Sci Rep. 2016 Dec 19;6:39253. doi: 10.1038/srep39253 (PMC5171786; doi:10.1038/srep39253)
Supplement: Supplementary Information [file srep39253-s1.pdf]

Supplementary Information

How mobile are dye adsorbates and acetonitrile molecules on the surface of TiO_2 nanoparticles? A quasi-elastic neutron scattering study

Valerie Vaissier,[1,5*] *Victoria Garcia Sakai,*[2] *Xiaoe Li,*[3] *Joao T. Cabral,*[4,5] *Jenny Nelson,*[1,5] *and Piers R. F. Barnes*[1,5*]

1. Department of Physcis, Imperial College London, London, SW72AZ, United Kingdom
2. ISIS Pulsed neutron and Muon Source, Rutherford Appleton Laboratory, Didcot, OX11 0QX, United Kingdom
3. Department of Chemistry, Imperial College London, London, SW72AZ, United Kingdom
4. Department of Chemical Engineering, Imperial College London, London, SW72AZ, United Kingdom
5. Centre for Plastics Electronics, Imperial College London, SW72AZ, United Kingdom

*vaissier@mit.edu, piers.barnes@imperial.ac.uk

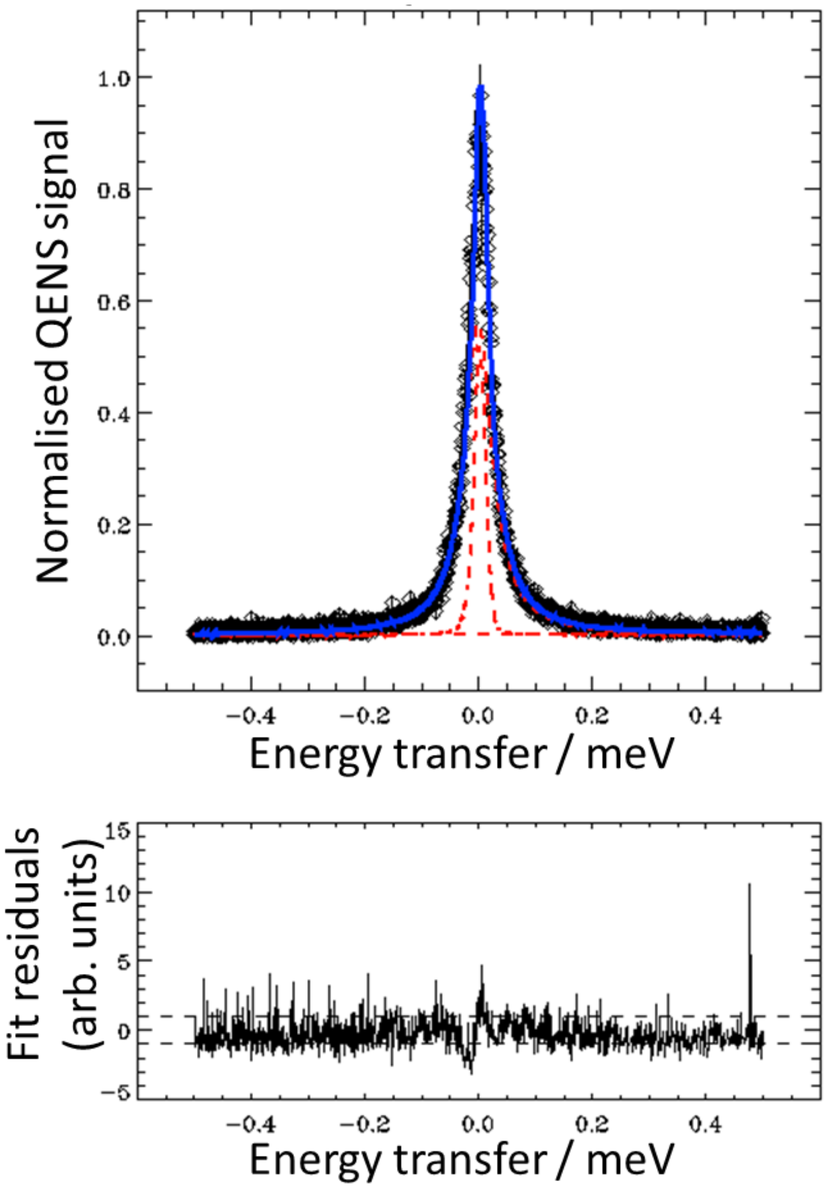

Figure S1. Example of a reconvolution fit to the QENS data (black lines with open diamonds) for detector group 1 ($Q \approx 0.25$ Å^{-1}) of the hACN sample (upper panel). Three components of the model are summed to give the total fit (solid blue line). The fitted model contains a negligible background contribution (horizontal dotted red line), an elastic component broadened by the instrument response (distinct dotted red peak), and a Lorenzian broadening of the signal (dotted red line, almost superimposed by the solid blue line). The residuals of the fit are shown in the lower panel.

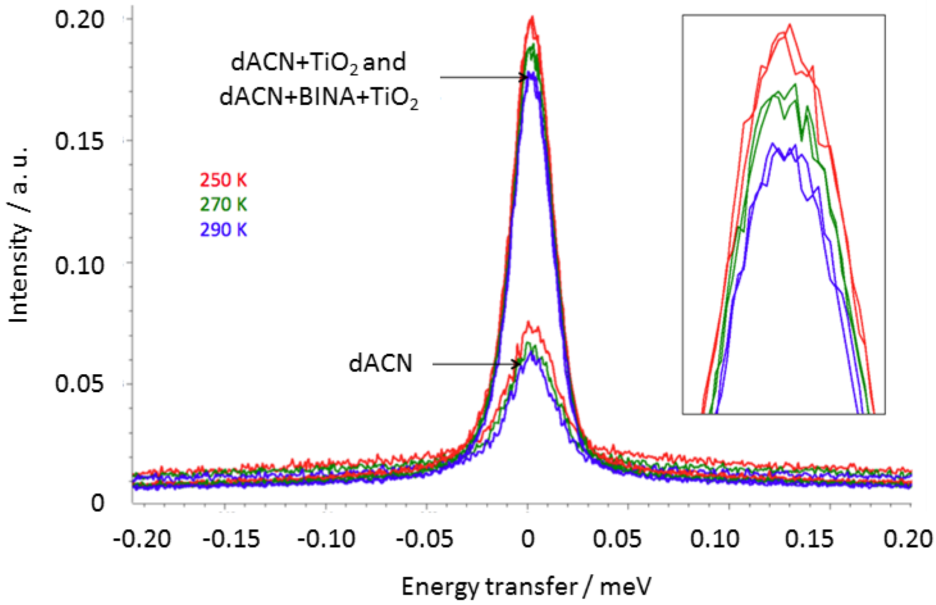

Figure S2. QENS spectra of dACN+BINA+TiO_2, dACN +TiO_2 and dACN at 250 K (red), 270 K (green) and 290 K (blue). The data are a sum of scattering for all Q-groups. The inset on the right hand side shows the peaks dACN+BINA+TiO_2, dACN +TiO_2 and dACN for each temperature. The same colour was used for both samples since no significant difference could be discerned between the samples with and without BINA.

Table S1. Acetonitrile diffusion coefficients, *D*, and time constants at high *Q* for rotational motion.

	**hACN**		**hACN+TiO2**	
**T** (K)	***D*** ($\times 10^{-5}$ cm^2 s^{-1})	***τ*** (ps)	***D*** ($\times 10^{-5}$ cm^2 s^{-1})	***τ*** (ps)
250	2.41±0.01	2.07±0.06	2.25±0.01	2.47±0.09
270	3.12±0.01	1.68±0.09	2.80±0.02	2.04±0.12
290	3.95±0.03	2.02±0.21	3.75±0.02	1.77±0.11

Errors correspond to fitting uncertainty. Note that jump diffusion models did not provide as good agreement as diffusion + rotation. We have defined the rotational time constant as $\tau = 2\hbar/\text{FWHM}$. The result for T = 290 K of τ = 1.8 ps agrees reasonably with the rotational time constant derived from the QENS peak width found in reference [1] where $\tau = 2\times0.658$ meV ps $rad^{-1}/(2\times0.590$ meV) = 1.1 ps rad^{-1}, and the time constant derived from NMR measurements[2] of $\tau = 1/(2D_{rotation})$ = 3.7 ps rad^{-1}. The values can also be compared to the rotational correlation times from a range of early simulations and measurements by a variety of techniques in table 1 of reference [3].

Table S2. Nominal sample compositions and relative scattering contributions.

Sample	Component:	hACN	dACN	TiO_2	INA	BINA
hACN	mass fraction	1.00				
(hACN: CH_3CN)	incoherent scattering fraction	0.90				
	coherent scattering fraction	0.10				
	**total scattering fraction**	**1.00**				
dACN	mass fraction		1.00			
(dACN: CD_3DN)	incoherent scattering fraction		0.15			
	coherent scattering fraction		0.85			
	**total scattering fraction**		**1.00**			
TiO_2	mass fraction			1.00		
	incoherent scattering fraction			0.22		
	coherent scattering fraction			0.78		
	total scattering fraction			1.00		
hACN+TiO_2	mass fraction	0.50		0.50		
	incoherent scattering fraction	0.88		0.01		
	coherent scattering fraction	0.10		0.02		
	**total scattering fraction**	**0.98**		**0.02**		
dACN+TiO_2	mass fraction		0.50	0.50		
	incoherent scattering fraction		0.13	0.07		
	coherent scattering fraction		0.74	0.06		
	**total scattering fraction**		**0.87**	**0.13**		
TiO_2+INA	mass fraction			0.98	0.02	
(INA: $C_6H_4O_2N$)	incoherent scattering fraction			0.17	0.20	
	coherent scattering fraction			0.59	0.04	
	**total scattering fraction**		**0.76**	**0.24**		
TiO_2+BINA	mass fraction			0.98		0.02
(BINA: $C_{12}H_6O_4N_2$)	incoherent scattering fraction			0.12		0.17
	coherent scattering fraction			0.66		0.04
	**total scattering fraction**			**0.79**		**0.21**
TiO_2+BINA+dACN	mass fraction			0.39	0.60	0.01
	incoherent scattering fraction		0.11	0.04		0.04
	coherent scattering fraction		0.66	0.14		0.01
	**total scattering fraction**		**0.77**	**0.18**		**0.05**

determined using scattering cross-sections from reference [4].

Supplementary References

1 Kunz, W., Calmettes, P. & Bellissent-Funel, M. C. Dynamics of liquid acetonitrile at high frequencies. *J. Chem. Phys.* **99**, 2079-2082, DOI:10.1063/1.465273 (1993).

2 Bopp, T. T. Magnetic Resonance Studies of Anisotropic Molecular Rotation in Liquid Acetonitrile-d3. *J. Chem. Phys.* **47**, 3621-3626, DOI:10.1063/1.1712431 (1967).

3 Evans, M. Molecular dynamics and structure of liquid acetonitrile - a review and computer simulation. *J. Mol. Liq.* **25**, 149-175 (1983).

4 Sears, V. F. Neutron scattering lengths and cross sections. *Neutron News* **3**, 26-37, DOI:10.1080/10448639208218770 (1992).
